# Supplementary material for: Enhancement of Electrochemical Detection of Gluten with Surface Modification Based on Molecularly Imprinted Polymers Combined with Superparamagnetic Iron Oxide Nanoparticles
Source: Polymers (Basel). 2021 Dec 27;14(1):91. doi: 10.3390/polym14010091 (PMC8747701; doi:10.3390/polym14010091)
Supplement: Supplementary file 1 [file polymers-14-00091-s001.zip › polymers-1480653-supplementary.pdf]

polymers

Supporting information

Enhancement of Electrochemical Detection of Gluten with Surface Modification Based on Molecularly Imprinted Polymers Combined with Superparamagnetic Iron Oxide Nanoparticles

Dalawan Limthin [1], Piyawan Leepheng [1], Annop Klamchuen [2] and Darinee Phromyothin [1,*]

[1] College of Materials Innovation and Technology, King Mongkut's Institute of Technology Ladkrabang, Bangkok 10520, Thailand; 63607009@kmitl.ac.th (D.L.); mildpiyawan55@gmail.com (P.L.)
[2] National Nanotechnology Center, National Science and Technology Development Agency, Patumthani 12120, Thailand; annop@nanotec.or.th
* Correspondence: darinee.ph@kmitl.ac.th

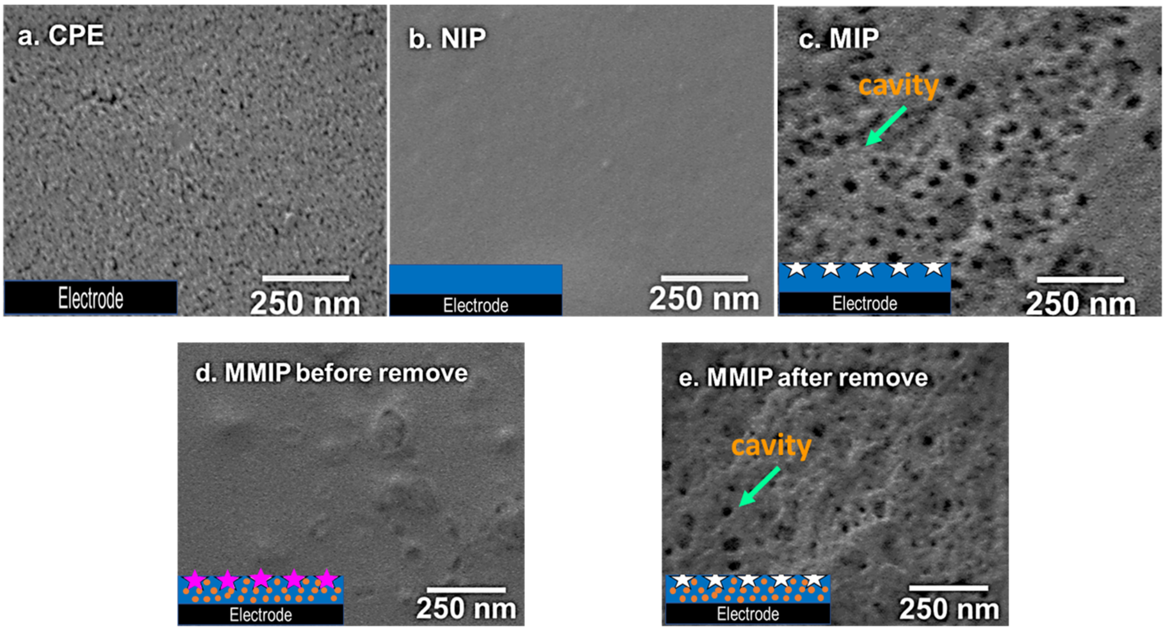

Figure S1. FESEM of surface-modified electrode consist of CPE, NIP, MIP, MMIP before and after remove.

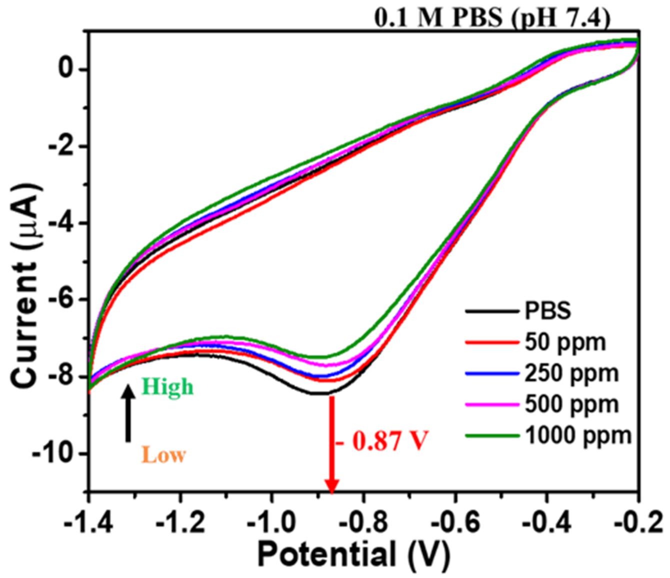

Figure S2. Cyclic voltammetry of MMIP modified electrode.

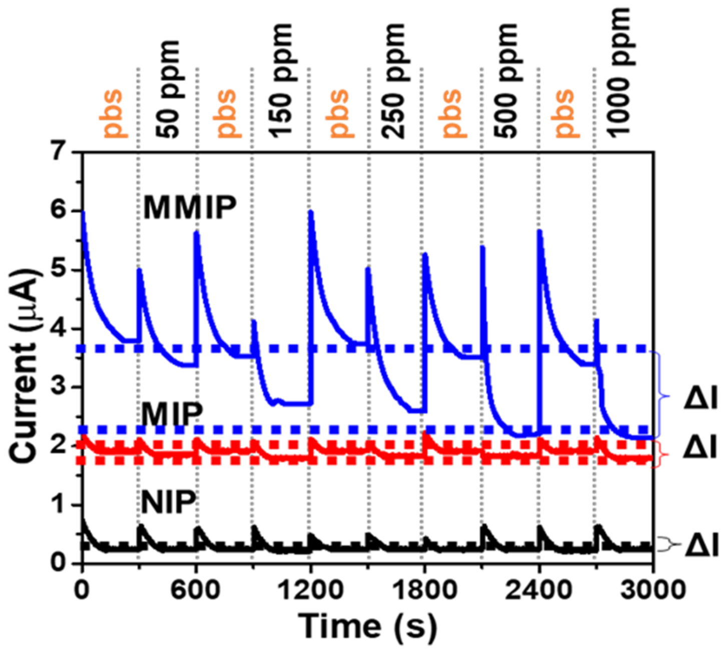

Figure S3. Amperometry of modified electrode.
